# Organoids as a Model for Colorectal Cancer

**DOI:** 10.1007/s11888-016-0335-4

**Published:** 2016-08-06

**Authors:** Madeleine Young, Karen R. Reed

**Affiliations:** European Cancer Stem Cell Research Institute, School of Biosciences, Cardiff University, Hadyn Ellis Building, Maindy Road, Cardiff, CF24 4HQ UK

**Keywords:** Colorectal cancer, Organoids, Intestinal stem cells, Cell culture, Modelling disease, CRISPR

## Abstract

Modelling human diseases in in vitro systems is undisputedly an invaluable research tool, yet there are many limitations. Some of those limitations have been overcome through the introduction of organoid culture systems, which have revolutionised colorectal cancer research and enabled an array of new experimental techniques. This 3D system models the physiology, shape, dynamics and cell make-up of the intestinal epithelium producing a relevant and highly adaptable model system. The increased functional relevance of this model compared to the use of 2D cancer cell lines makes it an invaluable tool for both basic and translational research. As the limitations of this system are being overcome to make high-throughput assays possible, it is clear that organoids are becoming a mainstay of colorectal cancer research. This review aims to explore the advantages and limitations of this system and discusses the future directions enabled by this model.

## Introduction

Colorectal cancer (CRC) is the fourth most common cancer type in the UK, but the second most common cause of death by cancer. Experimental systems in which the development of CRC can be modelled crucially underpin many advances in the development of novel therapies for CRC. However, despite increased understanding of the causes of CRC, the disease modelling systems available to researchers still remain limited.

The role of intestinal stem cells (ISCs) as the cell of origin of intestinal cancer has been well described and generally accepted [1], with the knowledge that it is mutations within the crypt base columnar (CBC) cells which drives initiation of tumourigenesis. In order to gain insight into the earliest stages of tumourigenesis, it is therefore necessary to study this population of cells.

Interestingly, there is still much dispute about which marker genes can reliably identify the CBC population [2] and the way in which stem cell dynamics contribute to CRC. The development of robust 3D culture systems from intestinal stem cells for the growth and maintenance of both normal and tumour tissue from intestinal and colonic samples of both mouse and human origin is already revolutionising the world of CRC research.

In 1992, a system for culturing 3D rat intestinal organoids was published [3, 4]. Despite providing a more functionally relevant culture system than previously available through the use of immortalised cell lines, the impact of this method was limited by the transient nature of the ISC divisions it enabled, resulting in established cultures only surviving for up to 1 month. In 2009, Sato et al. published a robust method which enabled the production of self-renewing intestinal organoids which could be expanded indefinitely [5]. The organoid culture method proposed by Sato in 2009 uses matrigel as a 3D matrix in which to embed either intestinal crypts or FAC-sorted Lgr5^+^ intestinal epithelium cells [5]. This method was soon optimised to enable the production of organoids from murine and human colon and colorectal adenoma tissue as well as from human colonic stem cells [6, 7].

In this organoid culture system, the epithelial cells are embedded within matrigel which supports growth within a 3D matrix, and the laminin-rich nature of matrigel mimics the microenvironment of the crypt base in vivo [8]. In addition, this system requires the provision of culture medium including growth factor supplements designed to support intestinal crypt growth and maintain the ISC population. Noggin (an inhibitor of the bone morphogenetic protein (BMP) signalling pathway) is added to the culture medium as BMP signalling has been shown to inhibit intestinal stem cell self-renewal [9]. Epidermal growth factor (EGF) is another key constituent of the growth medium due to its association with proliferation within the intestinal epithelium [10]. R-spondin is an addition to the culture medium required when growing organoids from normal or non-Wnt-activated intestinal epithelium. R-spondins are activators of the Wnt-signalling pathway which act specifically through the Lgr receptors (such as the ISC marker Lgr5) and thereby specifically upregulate Wnt-signalling within the ISC population, as well as driving crypt hyperplasia in vivo [11, 12]. In addition to these growth factors, the Rho-kinase inhibitor Y-27632 is required for culturing single cells, as it has been shown to inhibit anoikis in isolated embryonic stem cells [13]. Interestingly, organoids grown from human CRC samples often grow more successfully in the absence of niche factors, dependent on the mutational background of the samples and which signalling pathways are activated [14••, 15••].

This method not only produces 3D organoids, but those organoids have all of the epithelial cell types observed in the original system, with intestinal organoids even maintaining a functionally discrete “crypt” region and a conserved migration of the differentiated cells along a crypt-villus axis. This makes the organoid system a more functionally relevant model to in vivo response than traditional 2D culture methods, and provides the effective “missing link” for researchers between in vitro and in vivo studies.

**Fig. 1:**
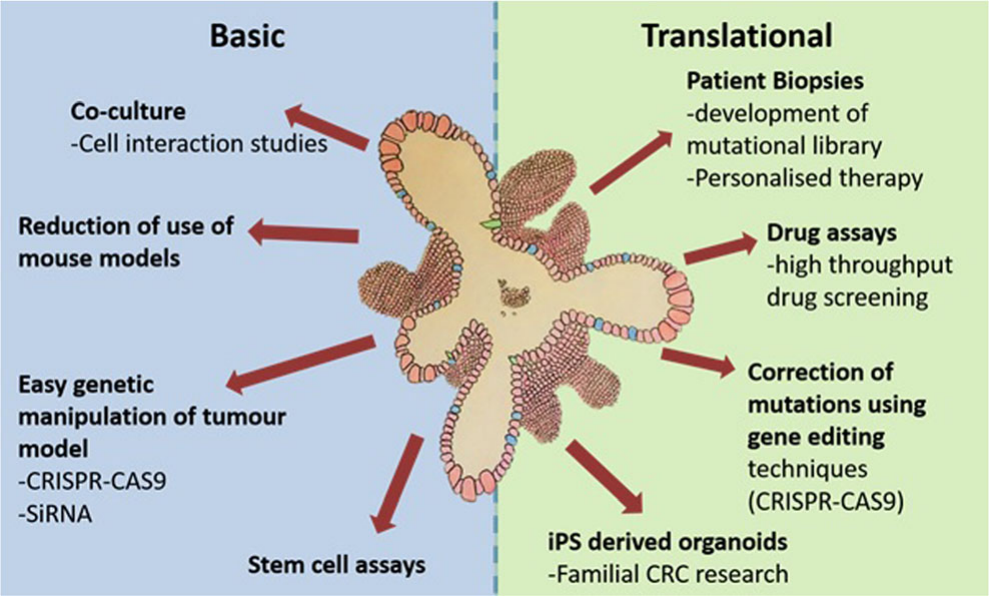
Organoid culture can be used as a model system for both basic and translational research

Modelling disease in vitro has many advantages over the use of animals in research. Aside from the obvious ethical advantage, in vitro modelling of colorectal cancer enables genetic alterations to be made easily, quickly and relatively cheaply. However, traditional 2D culture techniques have held numerous disadvantages due to cell homology and lack of cell/cell interactions. The use of 3D culture techniques such as those described above opens a brand new range of possibilities for modelling colorectal cancer, enabling the exploitation of a brand new toolkit in researching this disease. Broadly speaking, CRC research can be subdivided into two main categories: basic research and translational research; and the use of organoids as a model system is already proving its utility in both areas (Fig. 1).

### BASIC: Stem Cell Assay

Despite ISCs being thought to be the cell-of-origin of CRC [1], the difficulty of quantifying the ISC population within mouse models has been a limiting factor in many areas of CRC research. ISC markers can be used to visualise the ISC population although there is controversy over the most appropriate markers to use [2]. One of the major hurdles is that the majority of ISC markers, including the most commonly used marker Lgr5, are themselves Wnt targets. As much CRC research relies on the mis-regulation of Wnt signalling to mimic the human condition, this may have an impact on the expression levels of these markers without directly affecting the functionality of the ISC population. In this way, the use of intestinal organoids can be exploited for the study of the ISC population.

The main hallmarks of adult stem cells include the ability to self-renew as well as the potency to divide to produce the complete range of different cell lineages found within a tissue. The ability to form organoids in culture was first used as an assessment of the adult stem cell population within the nervous system [16], but has since been used as a stem cell assay within a variety of different tissues, including pancreas [17], mammary [18] and skin [19]. Given that it is known that Lgr5^+^ intestinal epithelial cells are the only cell type capable of efficiently forming differentiated organoids in culture, it is possible to apply the same methodology to the intestine [20, 21]. This has enabled a functional readout of “stemness” within the gut through the determination of organoid formation efficiency and organoid self-renewal efficiency. Interestingly, it was the ability to form organoids in culture which enabled the identification of human colonic stem cell population as the subset of colonic epithelial cells which express high surface levels of Ephb2 [7]. Thus, adaptation of different 3D culture systems is able to functionally assess the stem cell capacity of different cell populations.

### BASIC: Ease of Genetic Manipulation

The development of CRC is a multistep process, with the requirement for mutations in several different genes for the progression of benign polyp to malignant adenocarcinoma [22]. Modelling this process has previously proved to be a challenge in vivo, where temporal control of genetic mutations is possible through the use of techniques such as Cre-LoxP, but sequential deletion of a series of genes is often impractical. This has led to a knowledge gap as to the importance of the series step-wise mutations observed in the development of the disease. The use of the Cre-LoxP technology has been successfully applied to organoid culture to increase understanding of tumour progression [23•], but given that these systems still require the establishment of the organoids from a pre-existing in vivo model, they are limited by the requirement for breeding mice of the correct genotype for experimentation, which is not only time consuming and expensive but also has ethical implications.

Genetic manipulation of intestinal and colonic organoids for the purposes of CRC research using siRNA has been explored and is possible [24], but is limited by technical difficulty and inefficiency of transfection in 3D.

The recent advances in genetic engineering as a result of the development of the clustered regularly interspaced short palindromic repeats-CRISPR-associated 9 (CRISPR-Cas9) system are ideal for use in an organoid system. The CRISPR-Cas9 system utilises a prokaryotic adaptive immune system to enable the controlled replacement of sections of genes of interest with specific and pre-designed gene mutations in live cells with a low rate of off-target mutations [25]. The use of CRISPR-Cas9 technology in cancer biology is reviewed in detail elsewhere [26]. CRISPR-Cas9 is already beginning to revolutionise the way in which organoid culture can be used for CRC research. Matano et al. used the CRISPR-Cas9 method to genetically engineer organoids derived from normal human intestinal samples in an attempt to recreate the sequential establishment of driver mutations observed in the development of CRC [14••]. Interestingly, the successful introduction of each of the mutations (*APC*, *SMAD4*, *TP53*, *KRAS* and *PIK3CA*) was selectable by alterations to niche factors in the culture media (removal of Wnt/Rspo, noggin, nutlin3- and MDM2 inhibitor, EGF, and the addition of a Mek inhibitor, respectively). This method of selection for successfully engineered cells is not reliant on the use of antibiotics. Furthermore, these genetically modified organoids recapitulate the independency from niche factors that is observed in human CRC organoids, supporting its role as a model system.

This model was further exploited to explore the role of these mutations in invasion via the implantation of the genetically engineered organoids under the kidney subcapsule of mice [14••]. One of the major complications in treating CRC is metastasis to the liver, which reduces survival rates dramatically; however, understanding of the mutations which control invasion and metastasis is limited by the lack of an accurate model. Traditionally, these processes are difficult to investigate in mice, as tumour burden often results in death prior to metastasis. However, using the organoid method coupled with CRISPR-Cas9 technology to recapitulate tumour progression in culture, engineered organoids were successfully implanted into mice under the kidney subcapsule to observe which CRC driver mutations were essential for invasion and metastasis [14••]. Interestingly, it was observed that despite containing all driver mutations associated with tumour progression, the engineered organoids did not form invasive tumours or metastasise like the organoids derived from human CRC samples. This research implicates the importance of other factors such as genomic instability in invasive behaviour and would not have been possible without the use of the organoid system and the flexibility of genetic manipulation that it enables.

### BASIC: Co-Culture

One of the main advantages of using the organoid culture system over in vivo models for studying CRC is the ability to study the role of genetic mutations within the epithelium whilst removed from the range of variables such as stromal interactions and immune response. However, although this enables the examination of tumour/intestinal response to a range of genetic mutations, it is limited by lacking the complexity of interactions which are observed in vivo. In order to study the impact of different biological systems on the intestinal epithelium in a controlled environment, co-culture can be used. Over the recent years, there has been a drive for the modification of culture conditions to enable the co-culture of organoids from either intestinal or colorectal adenoma origin with a variety of different cell types including lymphocytes, nerves and fibroblasts [27–29]. These techniques, which vary from subtle alterations of current techniques [27, 29] to the use of well inserts [28], offer the opportunity to explore areas of CRC research that have until now been inaccessible.

### TRANSLATIONAL: Drug Assays

One of the greatest advantages of in vitro work over in vivo work is the ability to perform large-scale drug assays with relative ease and equally minimising the requirement for the use of animals for research. Previously, 2D cell culture has enable dramatic advances in drug screening for potential chemotherapeutics but was limited by the lack of cellular heterogeneity and cell/cell interactions observed in vivo, making results potentially unreliable. The use of immortalised cell lines in order to assess drug efficacy is in many ways limited, with nearly 95 % of the drugs which are found to have activity in 2D cell cultures failing to reach clinical trial [30]. It has been shown that intestinal crypt organoids are more accurate at predicting the apoptotic response to 5-FU in the intestinal epithelium of mouse models than either of the commonly used CRC cell lines Caco-2 or MC38 [31•]. In addition to this, it has been shown that 3D spheroids formed from pancreatic ductal adenocarcinoma develop chemoresistance in a more physiologically relevant manner than equivalent 2D culture [32]. As the development of chemoresistance is an important limiting factor in the utility of pharmacological intervention in CRC patients, this system may also provide a more functionally relevant model for CRC research. It is thought that 3D cell cultures provides a more accurate model than 2D cell culture because physical forces and spatial interactions between cells play an important role in many cellular signalling pathways [33].

Using ex vivo 3D primary culture as opposed to long-term cultured immortalised cell lines has advantages seen from many angles. In order to develop effective chemotherapeutics, the pharmaceutical industry must investigate two main areas: the efficacy of the drug at inducing apoptosis within tumour cells and the toxicity of the drug to untransformed cells. As one of the most common side effects of most chemotherapeutic treatments is gut toxicity, this is particularly important in the field of CRC research. In vitro modelling of both tumour and normal intestinal epithelium is frequently performed using the same system: transformed cell lines, making it difficult to differentiate between positive drug effect and negative side effects. The use of organoids enables the direct comparison between response in tumour spheroids and response in normal intestinal epithelial organoids, leading to a more informative readout of drug efficacy.

Through the use of more functionally relevant cell culture systems such as organoid culture, the efficacy of potential chemotherapeutics suitable for use in CRC patients can be explored prior to animal trials. This not only increases time efficiency but it also enables the abandonment of ineffective drugs prior to animal trials, resulting in a reduction in the use of animal models. Another major advantage of using this model system for drug assays is that organoids can be grown in a 96-well and even 384-well format. This enables multiple replicates using a wide range of samples and concentrations whilst only using low volumes of potentially expensive and difficult to acquire drugs.

One of the hurdles which is being overcome to enable the use of organoid culture in medium to high throughput drug studies is the issue of quantification. When healthy, organoids present a closed system with a clean, light appearance with clear epithelial integrity. However, when there is an increased number of dead or dying cells within the organoid, the outer surface is disrupted, displaying more loosely packed, often smaller cells and an overall darker and less uniform appearance, see Table 1. These “disrupted” organoids have been identified as representative of increased cell death [31•], but the difficulty remained with quantification. In order to count live/dead organoids in response to drug treatment, it was necessary to rely on manual observations using brightfield microscopy, a method which is not only labour intensive but also susceptible to inherent variations which arise from non-automated systems. One common route for quantifying cell death in traditional cell culture systems is through the use of MTT staining, whereby viable cells convert MTT into intracellular formazan, a purple dye detectable by spectrophotometry once solubilised using DMSO. As the 3D matrix in which the organoids are embedded (matrigel) is not soluble in DMSO, this technique had to be adapted for use in this system, by incorporating a 2 % SDS step prior to DMSO exposure in order to solubilise the matrigel [31•]. In addition to the quantification of cell death, drug efficacy can be assessed by measuring changes in cell viability. Commercially available reagents, such as CellTiter-Glo® and PrestoBlue®, have been widely used to assess metabolic activity and have been used with success in 3D organoid culture. CellTiter-Glo® is capable of quantifying ATP levels in a one-step end-point luminescence assay, whilst PrestoBlue® changes colour to become fluorescent in the presence of the reducing cytosol associated with living cells. One advantage of PrestoBlue® is that it does not require the disruption of the cells and so can be used as a live-cell assay, enabling re-use of the organoids.

**Table 1:** Identification of living wild-type intestinal organoids through defined morphological features

	Healthy/viable	Disrupted/non-viable
Brightfield microscopy		
Physical attributes	Closed system with epithelial integrity Ordered, tightly packed cells Defined crypt protrusions Identifiable luminal region	Absence of defined edge Disordered, smaller, diffuse cells No obvious structural elements Dark central region

Although these methods of assessing drug efficacy are useful, they are limited to assessing cell viability and do not assess other changes within the organoids. The structural complexity of organoids is an element of challenge not present when using 2D cell culture. For example, are the number of crypt-like protrusions remaining constant whilst undergoing treatment? Are the organoids smaller or larger? Are they made up of the same size cells? Each of these readouts may provide important data on the potential utility of proposed chemotherapeutic agents. In order to assess all of these many potential phenotypic changes, companies such as OcellO can screen 384 well plates of organoids using confocal imaging and specially designed software and image analysis to provide phenotypic profiles based on more than 900 different parameters [34, 35]. As these systems and techniques become more widely used, it is clear to see how high-throughput drug assays using organoids will become quicker, simpler and more informative.

### TRANSLATIONAL: Patient-Derived Organoids

An additional advantage of using organoids over in vivo studies is the ability to produce patient-derived organoids, either from tumour and tumour-associated normal tissue from patient biopsies, or from induced pluripotent stem (IPS) cells [36].

Culture of organoids derived from patient biopsies enables the thorough testing of a range of therapeutic compounds in a patient-relevant model. Although it is impossible to say that the organoids derived from these sources remain genetically and physiologically identical to the primary source, Jung et al. used comparative genomic hybridization analysis to conclude that the genomes of colorectal cancer stem cells which were cultured long term as 3D spheroids remained stable [7]. The ability to test a range of chemotherapeutics on actual patient-derived organoids could lead to patient-specific personalised therapy. On a broader scale, production of a range of patient -derived organoids from a variety of different genetic profiles will enable the exploration of specialised drug treatment plans post-biopsy, as well as enable genetic manipulation using technologies such as CRISPR-Cas9 to isolate the genetic mutations responsible for specific cancer phenotype [37].

The development of such a bank has already begun, with the establishment of a “living organoid biobank” of organoids derived from colorectal cancer patients [38]. By storing organoids derived from both malignant and normal tissue of the same patient, each sample has its own perfect control. As part of this biobank, the mutational signature of the original tumour biopsy and the organoid lines was assessed and compared. This enabled the analysis of drug response of groups of organoids with similar driving mutations, to assess drug utility. The large scale of this project meant that a high-throughput system was essential, and so drug screening systems similar to those previously used in 2D culture were utilised in a robotic system [39]. As this organoid bank grows, it is hoped that more associations between gene mutations and drug response will present themselves, making personalised therapy a more attainable goal for the treatment of CRC.

The growth of intestinal organoids from iPS cells yet another development of this system which could have huge impact on the world of CRC research [36]. By taking somatic cells of patients with familial adenomatous polyposis (FAP), producing iPS cells and directing differentiation into intestinal organoids, a model of FAP can easily be produced without the need for invasive biopsy. This is already being achieved with other forms of familial cancer [40] and could help aid the development of preventative therapies.

## Conclusions

Organoid culture is already making impacts on CRC research, and as the technique becomes more widely used, it is expected that it could provide the missing link between cell culture and in vivo modelling of the disease. By providing an easy-to-handle model in which genotype and phenotype can be directly compared in a short space of time, the use of organoid culture opens up an array of different experimental techniques which were previously not possible. However, in spite of the great utility of this model, the challenges of organoid culture should not be overlooked.

As previously mentioned, human CRC organoids have varied requirements for niche factors dependent on the pathways activated within the source tumour [14••, 15••]. As the growth factors which are added to the culture medium are selected to drive the most growth of patient-derived organoids, this may result in a bias towards the outgrowth of a particular subclone within the tumour, leading to differences in the genetic background between the tumour in vivo and ex vivo. The observation from Matano et al. that gene expression changes in organoids compared to in vivo as a result of progressive mutations was comparable, despite changes to growth factor exposure in culture, indicates that niche bias is not a problem in this system [14••]. However, it has been noted that under normal culture conditions (with all growth factors added), normal human colonic organoids outcompete CRC organoids [38], leading to the conclusion that some bias as a result of niche factors is possible, and must be considered when planning experiments.

As with any in vitro system, there is always a potential for genetic drift away from the original sample during long-term culturing and multiple passages. Despite the observation that CRC organoids remain genetically stable, there is no reason why organoids will not be at the same risk of genetic drift as 2D cell culture [41]. As patient-derived CRC organoids come from frequently genetically unstable and heterogeneous tumours, there must remain the possibility of genetic divergence from the original tumour. However, this risk can be reduced by limiting culture time and minimising passage number.

As with any model system, there are always limitations, and differences between the response of intestinal epithelium in vitro and in vivo are inevitable due to the absence of other cell types within organoid culture. For example, the absence of paneth cell-produced Wnts is tolerated in vivo, but not in organoid culture [42]. However, as was seen by the differences in metastatic potential between organoids genetically engineered to contain major driver mutations and organoids from genomically unstable CRC tumour samples [14••], these differences can hugely benefit our understanding of tumour development.

In spite of these potential differences, indeed often because of them, the utility of 3D organoid culture as a model for CRC research and the impact that this system is having on the field is clear.
